# Job Stress and Burnout Among School Health Teachers During the COVID-19 Pandemic: The Mediating Effect of Resilience and the Moderating Effect of School Organizational Culture

**DOI:** 10.3390/healthcare12222247

**Published:** 2024-11-11

**Authors:** Hye Ran Jung, Mi Heui Jang, Min Jung Sun

**Affiliations:** 1 Public Health Education, Graduate School of Education, Kyung Hee University, Seoul 02447, Republic of Korea; hrj252@korea.kr; 2 College of Nursing Science, Kyung Hee University, Seoul 02447, Republic of Korea; dnfntk0213@khu.ac.kr

**Keywords:** school health teacher, COVID-19, job stress, burnout, resilience, organizational culture

## Abstract

**Objectives:** This study aims to examine the mediating effect of resilience and the moderating effect of school organizational culture on the relationship between job stress and burnout among school health teachers during COVID-19. **Methods:** The participants of the study were 223 school health teachers. The data collected included the Korean version of the Connor-Davidson Resilience Scale (K-CD-RISC), Job Stress Scale, Maslach Burnout Inventory (MBI), and School Organizational Culture Scale. Data analysis was performed using SPSS/WIN 25.0 software. **Results:** There was a significant positive correlation between job stress and burnout among school health teachers. Conversely, both resilience and school organizational culture were negatively correlated with burnout. The mediating effect of resilience on the relationship between job stress and burnout was significant. However, the moderating effect of school organizational culture was not significant. **Conclusions:** To prevent burnout in school health teachers, it is necessary to develop policy alternatives aimed at reducing job stress and to implement psychological and emotional support measures to improve resilience.

## 1. Introduction

Since the initial reporting of COVID-19 cases in Wuhan, China, in December 2019, the world has been grappling with a pandemic. In response, most schools globally have adopted physical distancing measures to curb the spread of the virus and mitigate its impact. School nurses have played a pivotal role as leaders in providing health services aimed at preventing infections and promoting the health of staff, students, and their families within educational institutions [1,2]. In the Republic of Korea, school health teachers have taken on the responsibility of planning and coordinating responses to infectious disease crises [3]. Their duties include fever monitoring, providing preventive education, daily tracking and reporting of patient statuses, conducting epidemiological investigations, and collaborating with health authorities. These extensive responsibilities have led to increased job burnout and stress among school health teachers [4].

Job stress is a process in which stressors, stemming from unmet demands, resources, and capabilities in the work environment, lead to the development of psychological, behavioral, or physiological tension [5]. This type of stress diminishes an individual’s quality of life [6] and can lead to physical, mental, and social illnesses [7]. It also impairs personal concentration, attention, and decision-making abilities, thereby hindering job performance [8,9]. Consequently, it adversely affects the role of school health teachers in managing and promoting the health of staff and students within schools [5].

Burnout is defined as a state or symptom of physical, emotional, and mental exhaustion, coupled with cynicism, in response to organizational job stressors [10]. It does not manifest uniformly across all individuals in the same environment, as both personal characteristics and situational factors significantly influence its occurrence [11]. Personal factors that contribute to burnout include psychological aspects such as self-efficacy [12,13], resilience [14], and leadership skills [15]. Situational factors contributing to burnout encompass high workloads, low staffing levels [16], job demands [13], and the school environment [17].

Numerous studies have explored the psychological risks healthcare professionals and teachers face during unprecedented infectious disease crises like COVID-19, along with various strategies to address these challenges. However, there is a notable gap in research concerning the job stress and burnout experienced by school health teachers, who function as both educators and healthcare providers during such crises. Burnout, in particular, is influenced by a range of situational and personal factors, which results in significant individual variability [18]. Considering that an effective response to infectious disease disasters requires not only the competencies of school health teachers but also community cooperation and support, it is essential to conduct comprehensive research that addresses all these factors in the context of school health teachers’ job stress and burnout [18].

Resilience refers to a robust personal capacity to manage stress, characterized by a positive acceptance of change, harmonious relationships, goal-oriented control, and spiritual influence. It involves a dynamic process through which individuals develop mechanisms to cope with challenges [19,20]. Resilience forms the basis for personal growth, aiding in the overcoming of difficulties and stress [21]. Strengthening resilience can help reduce emotional exhaustion among nurses, boost work engagement, and improve problem-solving skills in the workplace [22]. Particularly during periods of ongoing uncertainty, such as the COVID-19 pandemic, resilience has been demonstrated to have a positive impact on psychological issues, including stress, anxiety, burnout, and fear [23,24]. Consequently, this study hypothesizes that resilience acts as a mediator in the relationship between job stress and burnout.

School organizational culture encompasses the shared philosophy, values, ideologies, beliefs, expectations, attitudes, norms, communication, and meaning systems among school members [25]. This culture influences both the shared values and behaviors of its members, thereby directly and indirectly impacting the responsibilities of school health teachers. During previous pandemics such as H1N1 and MERS, the importance of information sharing and the establishment of a cooperative response system within schools were highlighted. It was observed that successful management required not only additional personnel to support health teachers but also the active participation and cooperation of other staff members, along with a shift in parental perceptions [26,27]. Consequently, this study posits that school organizational culture may moderate the relationship between job stress, resilience, and burnout.

**Figure 1:**
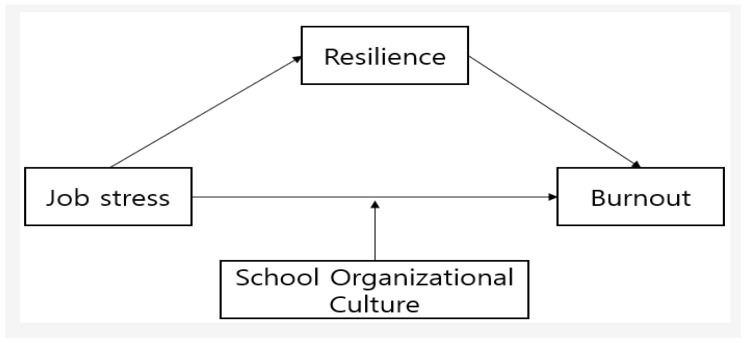
The conceptual framework of this study.

To date, numerous empirical studies have examined the status, experiences, roles, infection control practices, and knowledge of school health teachers during the COVID-19 pandemic. However, there has been a lack of research analyzing the levels of job stress and burnout among these educators, as well as the factors that influence these conditions. Therefore, this study aimed to identify the job characteristics related to COVID-19 that affect school health teachers during the pandemic and to assess their levels of burnout based on general and job-specific characteristics. Additionally, this research explored the correlations between job stress, burnout, resilience, and school organizational culture among school health teachers. It will also examine the mediating role of resilience and the moderating role of school organizational culture in the relationship between job stress and burnout. The conceptual framework of this study is illustrated in Figure 1.

## 2. Materials and Methods

### 2.1. Study Design and Participants

This descriptive correlational study was conducted to explore the mediating effect of resilience and the moderating effect of resilience and the moderating effect of school organizational culture on the relationship between job stress and burnout among school health teachers during the COVID-19 pandemic.

The selection criteria for participants were as follows: (i) school nurses with at least three years of experience working in elementary, middle, or high schools in the Republic of Korea and (ii) individuals who have responded to the COVID-19 pandemic over the past two years. Exclusion criteria included individuals who have taken a leave of absence (including for illness, childcare, or personal reasons) for more than six months since February 2020. These criteria were established considering previous research [28] showing that new school nurses with less than two years of experience or without prior experience as substitute school nurses face greater challenges in performing their duties, as well as the fact that the COVID-19 pandemic has persisted for over two years.

The appropriate sample size was determined using G*Power version 3.1. This calculation incorporated a medium effect size of 0.15, a significance level of 0.05, a power of 0.95, and 13 predictor variables, which included seven general characteristics, three job characteristics, job stress, resilience, and school organizational culture, as outlined in previous studies [29]. The initial calculation indicated a need for 189 participants. To account for a potential dropout rate of 20%, 227 participants were initially selected. After excluding four incomplete responses, data from 223 participants were ultimately used for the analysis.

### 2.2. Measures

The tools used in this study were utilized after obtaining permission from the relevant authors and institutions via e-mail following an explanation of the study’s purpose.

General Characteristics and Job-related Characteristics: This section included a total of nine items: age; highest educational attainment; teaching experience; marital status; school level (elementary, middle, or high); school size (number of students); workplace location (metropolitan or non-metropolitan); experience with COVID-19 patient transportation; and experience with COVID-19 contact tracing.

Job Stress: The tool developed by Jayaratne and Chess [30] was adapted by Yim and Kim [31] for use in the school context during the COVID-19 period. It comprises 15 items distributed across five subscales: role conflict (3 items), role ambiguity (3 items), workload (4 items), autonomy (3 items), and financial rewards (2 items). Responses were measured using a 5-point Likert scale, with higher scores indicating greater job stress. In Yim and Kim’s study [31], the tool’s reliability was shown by a Cronbach’s α value of 0.91, while, in this study, the Cronbach’s α was 0.86.

Burnout: The Maslach Burnout Inventory (MBI), developed by Maslach and Jackson [10], was adapted by Park [32] for use in this study. This scale consists of 18 items categorized into three subscales: emotional exhaustion (9 items), depersonalization (4 items), and reduced personal accomplishment (5 items). Responses are measured on a 5-point Likert scale, which ranges from “not at all” (1) to “very much” (5). Reverse scoring is utilized for items 7, 10, 13, 15, and 16, which assess reduced personal accomplishments. Higher scores reflect greater levels of burnout. The reliability of the scale was shown by a Cronbach’s α value of 0.79 in Park’s study [32], which improved to a Cronbach’s α value of 0.83 in the current study.

Resilience: The study employed the Korean version of the Connor-Davidson Resilience Scale (K-CD-RISC), which Baek adapted and standardized for Korean use [33]. This version is based on the original Connor-Davidson Resilience Scale (CD-RISC) developed by Connor and Davidson [19]. The scale comprises 25 items distributed across five subscales: hardiness (9 items), persistence (8 items), optimism (4 items), support (2 items), and spirituality (2 items). Responses are measured on a 5-point Likert scale ranging from “not true at all” (0) to “true nearly all of the time” (4), where higher scores denote greater resilience. The reliability of the tool was shown by a Cronbach’s α of 0.89 at the time of its development [33], and the Cronbach’s α was 0.95 in the current study.

School Organizational Culture: The study employed an instrument developed by Im [31], which modified the hospital organizational culture tool originally designed by Moon [34] into a school organizational culture tool for addressing COVID-19 infections in schools. This tool comprises nine items rated on a 5-point Likert scale, with higher scores reflecting a more positive perception of the school’s organizational culture. The items assess various aspects, including staff cooperation, adherence to COVID-19 guidelines, encouragement from school administrators, distribution of responsibilities by administrators, autonomy, responses by administrators to guideline violations, participation in prevention activities by members, cooperation in testing by members, and cooperation from students and parents. The reliability of the instrument was shown by a Cronbach’s α of 0.91 in Lim’s study [31], while the Cronbach’s α was 0.86 in the current study.

### 2.3. Procedure

This study was approved by the Institutional Review Board of Kyung Hee University (KHSIRB-22-557), and we requested remote cooperation from representatives of the National School Nurses Association community and local branches regarding the study. Subsequently, a recruitment notice was posted on the online bulletin board. Only participants who read the notice and consented to participate in the study were directed to the specified link to complete the survey. To prevent fraudulent responses in this online-based survey, all survey items were set as mandatory, and phone numbers collected prior to distributing rewards were checked for duplicates to prevent multiple submissions. The notice was posted from 26 January to 26 February 2022.

### 2.4. Statistical Analysis

The data for this study were analyzed using the SPSS WIN 25.0 program. We examined the general characteristics and job characteristics of the participants, as well as variables such as job stress, burnout, resilience, and school organizational culture. These were analyzed using means, standard deviations, the *t*-test, and ANOVA, with the Scheffe test employed for post hoc analysis. The relationships between job stress, burnout, resilience, and school organizational culture were assessed using Pearson’s correlation coefficient. To explore the mediating role of resilience and the moderating role of school organizational culture in the relationship between job stress and burnout, we employed Hayes’ PROCESS macro [35].

## 3. Results

### 3.1. General and Job Characteristics of the Study Participants

The average age of the participants was 39.31 ± 8.38 years, with the largest age group being those in their 30s, comprising 41.3%. Regarding educational attainment, 174 participants (78.0%) held a bachelor’s degree. The majority, 122 participants (54.7%), had less than 5 years of teaching experience, and 143 participants (64.1%) were married. In terms of workplace, 119 participants (53.4%) worked in elementary schools, 61 (27.4%) in middle schools, and 43 (19.3%) in high schools. The most common school size was between 100 and 500 students, involving 90 participants (40.4%), and 121 participants (54.3%) worked in the metropolitan area. Regarding experiences related to COVID-19, 165 participants (74.0%) had never transferred students or staff to screening clinics, while 33 participants (14.8%) had done so one to two times. In terms of conducting epidemiological investigations due to confirmed cases within the school, 67 participants (30.0%) had done so one to two times, followed by 54 participants (24.2%) who had done so three to five times (Table 1).

### 3.2. Levels of Job Stress, Burnout, Resilience, and School Organizational Culture

The levels of job stress, burnout, resilience, and school organizational culture among the participants are shown in Table 2. The average score for job stress was 3.82 ± 0.63, while the average score for burnout was 3.12 ± 0.75. Resilience had an average score of 2.60 ± 0.65, and the school organizational culture had an average score of 3.38 ± 0.74.

### 3.3. Differences in Burnout According to General and Job Characteristics of the Study Participants

The differences in burnout according to the participants’ general and job characteristics are shown in Table 1. Significant differences in burnout levels were noted with respect to age (F = 3.54, *p* = 0.016), school size (F = 2.97, *p* = 0.020), and the number of epidemiological investigations conducted due to confirmed cases (F = 2.71, *p* = 0.031). Post hoc analyses revealed that participants between the ages of 30 and 39 experienced higher levels of burnout compared to those aged 40–49. Additionally, participants working in schools with 500–699 students reported more burnout than those in schools with fewer than 100 students. Furthermore, individuals who had conducted more than 11 epidemiological investigations reported greater burnout than those who had conducted only 1–2 investigations.

### 3.4. Correlations Among Job Stress, Burnout, Resilience, and School Organizational Culture

The analysis of correlations among job stress, burnout, resilience, and school organizational culture in school health teachers identified several significant relationships. Strong positive correlations were found between burnout and job stress (r = 0.59, *p* < 0.001) and between resilience and school organizational culture (r = 0.49, *p* < 0.001). In contrast, job stress was negatively correlated with both resilience (r = −0.28, *p* < 0.001) and school organizational culture (r = −0.59, *p* < 0.001). Similarly, burnout was strongly negatively correlated with resilience (r = −0.45, *p* < 0.001) and school organizational culture (r = −0.48, *p* < 0.001) (Table 3).

### 3.5. Factors Influencing Burnout Among School Health Teachers

To identify the factors influencing burnout among school health teachers, a multiple regression analysis was conducted. This analysis accounted for variables such as age, school size, and the number of epidemiological investigations, which were significant in the general characteristics. The impact of job stress, resilience, and school organizational culture on burnout was also explored, and the results are presented in Table 4.

The Durbin–Watson statistic for burnout was 1.936, indicating independence (du = 1.913) and no autocorrelation. The variance inflation factor values ranged from 1.442 to 3.286; all values were below 10, suggesting that there were no multicollinearity issues among the independent variables.

The multiple regression analysis indicated that the burnout levels were significantly higher in schools with student populations between 500 and 700 (β = 0.21, *p* = 0.009), under conditions of elevated job stress (β = 0.47, *p* < 0.001), and when resilience was lower (β = −0.27, *p* < 0.001). The model explained 45.6% of the variance in burnout, with job stress contributing the most significant impact.

### 3.6. Mediating Effect of Resilience on the Impact of Job Stress on Burnout Among School Health Teachers

After controlling for age, school size, and the number of epidemiological investigations—as variables that demonstrated significant differences in burnout among the general characteristics—the significance of each pathway was evaluated, as shown in Table 5.

Job stress significantly impacted both resilience (B = −0.35, *p* < 0.001) and burnout (B = 0.59, *p* < 0.001). Additionally, resilience had a significant effect on burnout (B = −0.33, *p* < 0.001). These findings demonstrate that job stress, the independent variable, and resilience, the mediator, both significantly influenced burnout, the dependent variable. Resilience served as a partial mediator in this relationship. The explanatory power of resilience’s mediating effect was 10.3%.

**Figure 2:**
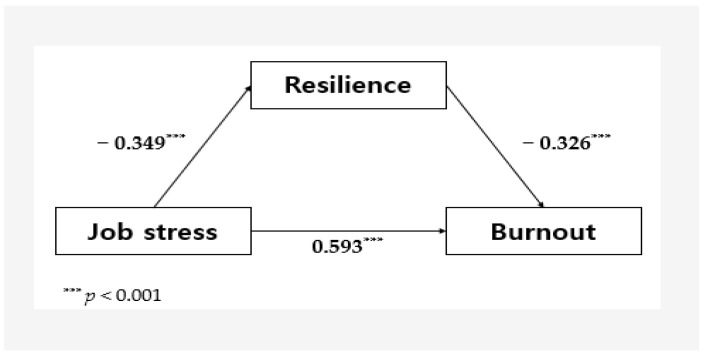
Mediating effect of resilience in the relationship between job stress and burnout.

A mediation analysis employing the PROCESS macro with bootstrapping confirmed that resilience significantly mediates the relationship between job stress and burnout (B = 0.71, 95% CI = 0.57–0.84). Figure 2 presents a diagram illustrating the mediating effect of resilience on the impact of job stress on burnout.

### 3.7. Moderating Effect of School Organizational Culture on the Impact of Job Stress on Burnout Among School Health Teachers

The results of the analysis testing the moderating effect of school organizational culture on the relationship between job stress and burnout among school health teachers are presented in Table 6. After adjusting for significant variables such as age, school size, and the number of epidemiological investigations, the moderating variable—school organizational culture—did not significantly influence burnout (B = 0.043, *p* = 0.894). Additionally, the interaction between job stress and school organizational culture did not significantly impact burnout (B = −0.024, *p* = 0.751).

## 4. Discussion

This study was conducted to explore the mediating effect of resilience and the moderating effect of resilience and the moderating effect of school organizational culture on the relationship between job stress and burnout among school health teacher during the COVID-19 pandemic.

The burnout level of school health teachers in this study averaged 3.12 points, which is higher than the average score of 2.74 for school health teachers before COVID-19, as measured by the same tool [32]. Since this study did not investigate the participants’ burnout levels before and after COVID-19, a direct comparison is not possible. However, it can be inferred that the pandemic has led to an increase in teachers’ burnout levels, in line with previous research [36]. This rise in burnout is likely due to the diverse and excessive workload they faced during the pandemic, which included managing infectious diseases at schools, conducting disinfection and epidemiological investigations, establishing response systems, and monitoring both staff and students. Additionally, the ongoing stress from an uncertain future and pandemic-related anxiety likely contributed to this increased burnout [37,38].

In this study, job stress, resilience, and school organizational culture were identified as significant factors influencing burnout among school health teachers. The findings revealed that higher levels of job stress correlated with increased burnout, whereas greater resilience and a more positive school organizational culture were linked to reduced burnout. These results are consistent with prior research, which has shown that job stress contributes to burnout among teachers and that resilience helps mitigate burnout in both nurses and teachers [39,40,41]. Additionally, the data support the possibility that a supportive school organizational environment is associated with lower burnout levels [42]. Therefore, to alleviate burnout among school health teachers, it is necessary to reduce job stress, enhance resilience, and promote a positive school organizational culture. This underscores the importance of developing targeted strategies to address burnout within these specific contexts.

A key finding of this study is that resilience partially mediated the relationship between job stress and burnout among school health teachers during the COVID-19 period. Specifically, job stress significantly impacts resilience; lower job stress is associated with higher resilience, which, in turn, leads to reduced burnout. This finding is consistent with previous research indicating that resilience mediates the relationship between job stress and burnout among teachers. Additionally, studies have shown that job stress and burnout are directly related, with resilience playing an indirect mediating role, especially among nurses responding to COVID-19 [43,44].

The global pandemic of COVID-19 frequently led to burnout among school health teachers due to excessive workloads, sacrifices, and anxiety [37,38]. However, overcoming these challenges through resilience can increase job satisfaction and enhance personal capabilities, providing opportunities to demonstrate greater professionalism [45]. Previous research has shown that various programs, including mindfulness training for teachers [46], self-care programs for nurses [47], and resilience programs [48], significantly enhance resilience and reduce burnout. Based on these findings, there is a clear need to develop and implement programs to strengthen resilience specifically tailored for school health teachers in the future.

In this study, the moderating effect of school organizational culture on the relationship between job stress and burnout was not significant. This indicates that school organizational culture did not positively influence the reduction of burnout. This finding is in contrast to previous research [49], which suggested that school organizational culture mitigates burnout and moderates job stress. The discrepancy may stem from the instrument used in this study, which was adapted from a tool originally designed for hospital organizational culture to fit school settings. It primarily assessed cooperation among staff, students, and parents regarding the responsibilities of school health teachers during the COVID-19 situation. However, it did not consider the school’s overall educational policies, work processes, adaptability to change, cohesion, and hierarchy awareness.

In response to infectious diseases such as COVID-19, it is crucial to have a collaborative system among organizational members. However, no tool is currently available to measure the role of school organizational culture in response to infectious diseases. Therefore, future efforts should concentrate on conducting in-depth analyses and developing measurable tools to assess the impact of school organizational culture in the context of infectious disease responses.

There is a possibility that selection bias occurred in this study, since participants voluntarily enrolled through the website. Future studies are expected to support these results with more diverse samples. Additionally, the school infectious disease prevention and management manual was revised over six times in line with the government’s quarantine guidelines during the COVID-19 pandemic. Consequently, job characteristics may vary depending on the period of revision, warranting further research tailored to these specific times. Finally, further research is essential for developing and evaluating resilience enhancement programs aimed at reducing burnout among school nurses during disaster situations, such as pandemics.

## 5. Conclusions

This study aimed to explore job stress and burnout among school health teachers during the COVID-19 period, analyzing the impacts of resilience and school organizational culture. The objective was to develop practical strategies to prevent teacher burnout in future responses to school infectious diseases. The findings suggest that the increased job stress experienced by school health teachers during the COVID-19 pandemic might be linked to higher levels of burnout. Conversely, greater resilience and a more positive school organizational culture might have been correlated with reduced burnout. Resilience was found to have a partial mediating effect on the relationship between job stress and burnout. However, the evaluation of school organizational culture, which only assessed the responsibilities of school nurses and cooperation among school personnel, did not significantly contribute to reducing burnout in this context. Therefore, further consideration of school organizational culture related to educational policies and work processes is necessary.

To prevent burnout among school health teachers during prolonged infectious disease crises, it is essential to offer additional support for their workload, increase staffing for infectious disease responses, and implement policies that address real-world challenges. Additionally, as a means of mitigating burnout, there is a need to develop and evaluate programs designed to boost resilience.

## Figures and Tables

**Table 1:** Differences in burnout according to general and job characteristics (*N* = 223).

Variables	Categories	*n*(%)	Burn Out	
			M ± SD	t or F(p) Scheffe
Age (years)	<30	23(10.3)	3.02 ± 0.73	3.54 (0.016) b > c
	30–39	92(41.3)	3.31 ± 0.70	
	40–49	78(35.0)	2.97 ± 0.92	
	≥50	30(13.5)	3.00 ± 0.87	
Education level	Associate degree	14(6.3)	3.00 ± 0.47	0.19 (0.831)
	Bachelor’s degree	174(78.0)	3.12 ± 0.76	
	≥Master’s degree	35(15.7)	3.13 ± 0.76	
Marital status	Single	80(35.9)	3.21 ± 0.69	1.39 (0.165)
	Married	143(64.1)	3.06 ± 0.77	
Type of school	Elementary school	119(53.4)	3.12 ± 0.84	0.04 (0.958)
	Middle school	61(27.4)	3.12 ± 0.60	
	High school	43(19.3)	3.09 ± 0.67	
Years of working in school (years)	<5	122(54.7)	3.16 ± 0.70	0.64 (0.635)
	5–9	46(20.6)	3.19 ± 0.80	
	10–14	13(5.8)	2.95 ± 0.71	
	15–19	7(3.1)	3.39 ± 0.71	
	≥20	35(15.7)	3.02 ± 0.85	
School size (persons)	<100	22(9.9)	2.73 ± 0.78	2.97 (0.020) c > a
	100–499	90(40.4)	3.04 ± 0.75	
	500–699	40(17.9)	3.32 ± 0.72	
	700–999	39(17.5)	3.26 ± 0.68	
	≥1000	32(14.3)	3.16 ± 0.73	
Workplace	Metropolitan	121(54.3)	3.26 ± 0.67	−1.75 (0.083)
	Non-metropolitan	102(45.7)	2.95 ± 0.76	
Experience of transfer to screening clinic	None	165(74.0)	3.11 ± 0.75	1.48 (0.222)
	1–2	33(14.8)	3.00 ± 0.73	
	3–5	13(5.8)	3.14 ± 0.73	
	≥6	12(5.4)	3.52 ± 0.65	
Experience of conducting epidemiological investigations	None	42(18.8)	3.02 ± 0.83	2.71 (0.031) e > b
	1–2	67(30.0)	2.93 ± 0.80	
	3–5	54(24.2)	3.25 ± 0.62	
	6–10	20(9.0)	3.13 ± 0.79	
	≥11	40(17.9)	3.35 ± 0.63	

**Table 2:** Levels of job stress, burnout, resilience, and school organizational culture (N = 223).

	Range	Mean ± SD	Min	Max
Job stress	1–5	3.82 ± 0.63	1.80	3.82
Burnout	1–5	3.12 ± 0.75	1.00	3.12
Resilience	0–4	2.60 ± 0.65	0.48	2.60
School organizational culture	1–5	3.38 ± 0.74	1.56	3.38

**Table 3:** Descriptive statistics and correlations of job stress, burnout, resilience, and school organizational culture.

	M ± SD	Job Stress	Burnout	Resilience	School Organizational Culture
Job stress	3.82 ± 0.63	1			
Burnout	3.12 ± 0.75	0.59 ***	1		
Resilience	2.60 ± 0.65	−0.28 ***	−0.45 ***	1	
School organizational culture	3.38 ± 0.74	−0.59 ***	−0.48 ***	0.49 ***	1

*** *p* < 0.001.

**Table 4:** Factors affecting burnout *(N* = 223).

		B	SE	β	t	*p*
(constant)		1.56	0.49		3.17	0.002
Age (years)	30–39	0.16	0.13	0.11	1.23	0.221
	40–49	0.08	0.14	0.05	0.61	0.545
	≥50	0.19	0.16	0.09	1.16	0.249
School size (persons)	100–499	0.23	0.14	0.15	1.71	0.090
	500–699	0.41	0.16	0.21	2.62	0.009
	700–999	0.29	0.16	0.15	1.82	0.071
	≥1000	0.21	0.17	0.10	1.27	0.207
Experience of conducting epidemiological investigations	1–2	−0.12	0.11	−0.07	−1.05	0.296
	3–5	0.06	0.13	0.03	0.47	0.636
	6–10	0.03	0.16	0.01	0.17	0.867
	≥11	0.17	0.13	0.09	1.29	0.198
Job stress		0.56	0.08	0.47	7.24	<0.001
Resilience		−0.30	0.07	−0.27	−4.46	<0.001
School organizational culture		−0.06	0.07	−0.06	−0.81	0.421
F(*p*) *R*^2^*(adj R*^2^*)* *d(d*_u_*)*		14.314(<0.001) 0.491(0.456) 1.936(1.913)				

**Table 5:** Mediating effect of resilience in the relationship between job stress and burnout.

		Step 1				Step 2			
		Resilience				Burnout			
		B	SE	t	*p*	B	SE	t	*p*
(constant)		4.17	0.33	12.60	<0.001	1.32	0.39	3.39	0.001
Age (years)	30–39	−0.19	0.15	−1.31	0.193	0.16	0.13	1.21	0.227
	40–49	−0.22	0.15	−1.48	0.140	0.07	0.14	0.54	0.590
	≥50	−0.45	0.18	−2.51	0.013	0.18	0.16	1.11	0.267
School size (persons)	100–500	−0.12	0.15	−0.78	0.438	0.23	0.14	1.69	0.092
	500–700	0.10	0.18	0.58	0.566	0.41	0.16	2.60	0.010
	700–1000	−0.20	0.18	−1.11	0.269	0.28	0.16	1.77	0.078
	>1000	−0.20	0.19	−1.07	0.287	0.21	0.17	1.23	0.220
Experience of conducting epidemiological investigations	1–2	0.21	0.13	1.68	0.095	−0.11	0.11	−1.01	0.313
	3–5	0.08	0.14	0.60	0.551	0.06	0.12	0.48	0.634
	6–10	−0.07	0.18	−0.36	0.719	0.04	0.16	0.24	0.811
	≥11	−0.03	0.15	−0.17	0.864	0.18	0.13	1.32	0.187
Job stress		−0.45	0.07	−4.89	<0.001	0.59	0.07	8.87	<0.001
Resilience						−0.33	0.06	−5.32	<0.001
F(*p*)		3.12(<0.001)				15.39(<0.001)			
R^2^(*adj* R^2^)		0.15(0.10)				0.49(0.46)			
*d(d_u_)*		2.03(1.89)				1.95(1.90)			

**Table 6:** Moderating effect of school organizational culture in the relationship between job stress and burnout.

		Step 1				Step 2			
		Resilience				Burnout			
		B	SE	t	*p*	B	SE	t	*p*
(constant)		4.17	0.33	12.60	<0.001	1.20	1.26	0.95	0.342
Age	30–39	−0.19	0.15	−1.31	0.193	0.16	0.13	1.23	0.220
	40–49	−0.22	0.15	−1.48	0.140	0.08	0.14	0.61	0.543
	≥50	−0.45	0.18	−2.51	0.013	0.18	0.16	1.12	0.265
School size (persons)	100–500	−0.12	0.15	−0.78	0.438	0.23	0.14	1.70	0.091
	500–700	0.10	0.18	0.58	0.566	0.41	0.16	2.30	0.010
	700–1000	−0.20	0.18	−1.11	0.269	0.29	0.16	1.80	0.073
	>1000	−0.20	0.19	−1.07	0.287	0.21	0.17	1.26	0.209
Experience of conducting epidemiological investigations	1–2	0.21	0.13	1.68	0.095	−0.12	0.11	−1.05	0.293
	3–5	0.08	0.14	0.60	0.551	0.06	0.13	0.48	0.634
	6–10	−0.07	0.18	−0.36	0.719	0.03	0.16	0.17	0.865
	≥11	−0.03	0.15	−0.17	0.864	0.17	0.13	1.28	0.201
Job stress		−0.45	0.07	−4.89	<0.001	0.65	0.30	2.21	0.028
Resilience						−0.31	0.07	−4.46	<0.001
School organizational culture						0.04	0.32	0.13	0.894
Job stress × school organizational culture						−0.02	0.08	−0.32	0.751
F(*p*)		3.12(<0.001)				13.31(<0.001)			
R^2^		0.151				0.491			

## Data Availability

The raw data supporting the conclusions of this article will be made available by the authors upon reasonable request after signing a confidentiality agreement.
